# Intestinal microbiota dysbiosis in acute kidney injury: novel insights into mechanisms and promising therapeutic strategies

**DOI:** 10.1080/0886022X.2022.2056054

**Published:** 2022-03-29

**Authors:** Juan Lei, Yifan Xie, Jingyi Sheng, Jiayu Song

**Affiliations:** a Department of Pediatric Nephrology, The Second Affiliated Hospital of Nanjing Medical University, Nanjing, People’s Republic of China; b Department of Rheumatism and Immunology, Children’s Hospital of Nanjing Medical University, Nanjing, People’s Republic of China

**Keywords:** Intestinal microbiota, kidney diseases, AKI, gut–kidney axis, intestinal microbiome dysbiosis, therapies

## Abstract

In recent years, the clinical impact of intestinal microbiota–kidney interaction has been emerging. Experimental evidence highlighted a bidirectional evolutionary correlation between intestinal microbiota and kidney diseases. Nonetheless, acute kidney injury (AKI) is still a global public health concern associated with high morbidity, mortality, healthcare costs, and limited efficient therapy. Several studies on the intestinal microbiome have improved the knowledge and treatment of AKI. Therefore, the present review outlines the concept of the gut–kidney axis and data about intestinal microbiota dysbiosis in AKI to improve the understanding of the mechanisms of the intestinal microbiome on the modification of kidney function and response to kidney injury. We also introduced the future directions and research areas, emphasizing the intervention approaches and recent research advances of intestinal microbiota dysbiosis during AKI, thereby providing a new perspective for future clinical trials.

## Introduction 

Acute kidney injury (AKI) is a major and life-threatening kidney disease resulting from pathogenic conditions, such as hemodynamic instability, sepsis, and drug toxicity [1,2]. The current understanding of AKI involves alteration of the microcirculation, renal tubular epithelial cell (RTEC) injury, and intrarenal inflammation. AKI causes severe complications with uremic toxins building up in the body, resulting in a decline of renal function [3]. Furthermore, AKI has been recognized as a non-self-limited process strongly linked to an increased risk of chronic kidney disease (CKD) [4]. However, no therapeutic strategies reliably improve survival except renal replacement therapies (long-term dialysis and kidney transplantation). However, renal replacement presents inherent disadvantages such as side effects, high cost, and increased risk of infections [5].

In the past few years, intestinal microbiome has gained increasing attention with respect to human health and diseases. Intestinal microbiota composition is in a certain proportion after birth, with beneficial bacteria as predominant species, thus forming a symbiosis of quality and quantity. Reportedly, intestinal microbiota dysbiosis is associated with colorectal cancer, liver diseases, obesity, and renal diseases [6–9]. A recent study showed changes in intestinal microbiota composition in animal models of ischemic AKI [10]. Increasing evidence suggested that intestinal microbiota and its metabolites contribute substantially to the pathogenesis of AKI [11]. A permanent alteration in the microbiota composition or function alters intestinal motility, and permeability initiates the immune response, thus improving visceral adaptivity [12,13]. In this review, we focused on the potential mechanisms of intestinal microbiota dysbiosis in AKI and summarized the recent research progress and promising therapeutic strategies.

## Role of the microbiota in gut–kidney axis

The human intestinal tract, especially the large intestine, harbors an extremely diverse population of microorganisms, termed intestinal microbiota. The human endogenous intestinal microbiota is recognized as an essential ‘organ’ in healthy individuals with a dynamic and beneficial association with the human host [14]. The most dominant bacterial phyla of the intestinal microbiota are *Firmicutes* and *Bacteroidetes*, while *Proteobacteria*, *Actinobacteria*, *Fusobacteria*, and *Verrucomicrobia phyla* are in the minority [15]. The intestinal microbiota composition of humans changes continuously over the life of the host, based on lifestyle, including eating habits, age, administration of antibiotics, and presence or absence of diseases [16]. The balance of the intestinal microbiota is essential for the integrity of the intestinal epithelial barrier [17], executing energy metabolism [18,19], and regulating immune health [20]. It also promotes the maturation of the intricate enteric nervous system (ENS) [21]. Colonization of germ-free (GF) mice with microbiota from conventional specific pathogen-free (SPF) mice releases 5-HT and activates 5-HT4 receptors, which in turn alter the neuroanatomy of the ENS and improve the rate of intestinal transit [22].

Recently, the gut–kidney axis has gained increasing attention, but the studies have mainly focused on CKD [23]. The basic principle of the gut–kidney axis is the crosstalk between the imbalance of intestinal microbiota and the decreased renal function [24]. In CKD, renal injury causes the accumulation of metabolic waste in the body, which could infiltrate into the intestinal cavity, causing intestinal microbiota dysbiosis. In addition, the intestinal epithelial barrier is impaired, facilitating the passage of opportunistic pathogens and enterogenic urinary toxins into the blood circulation, which triggers a systemic inflammatory response and deteriorates renal disease [25,26]. Furthermore, intestinal dysbiosis and its metabolites play a critical role in blood pressure control. For example, Nα-acetyl-L-arginine, stearic acid, phosphatidic acid, and glucoside are abundant in hypertension samples [27]. Similarly, metabolomics illustrated that gut microbial metabolites are involved in the pathogenesis and progression of diabetic kidney disease (DKD), including short-chain fatty acids (SCFAs), trimethylamine-N-oxide (TMAO), bile acids (BAs), polyphenols, tryptophan-derived metabolites, branched-chain amino acids (BCAAs), and other metabolites [28]. However, intestinal microbiome dysbiosis is also crucial in AKI-to-CKD transition. Changes in the intestinal microbiota associated with these metabolites are also detected in early kidney disease [29]. Such as, SCFAs were shown to decrease the inflammatory response, reduce the infiltration of damaged tissue by leukocytes and affect chemotaxis and cytokine production in a murine IRI model of AKI.

Interestingly, renal tubular damage is also frequently observed in patients with inflammatory bowel disease (IBD), and the morbidity of IBD-related renal manifestations is up to 20% [30]. In addition, intestinal immune tolerance deficiencies induce the absorption of antigens and activate the mucosal lymphoid tissue (MALT), causing excessive deposition of abnormal IgA1 in the glomerular region, eventually developing IgA nephropathy (IgAN) [31]. Recent advancements in basic and clinical research have demonstrated that dysfunction of other organs underlies the poor outcomes of AKI [32]. Significant progress has been made in renal therapy based on the gut–kidney axis, including modulation of the gut environment, improvement of the systemic inflammatory response, and decline in toxin accumulation; together, these factors reverse the renal injury [33,34].

## Intestinal microbiota and AKI

Recent evidence from studies on AKI-to-CKD transition and end-stage renal disease (ESRD) indicated that changes in intestinal microbiota occur in kidney diseases [35]. A previous study on AKI has focused on the analysis of intestinal microbiota composition by sequencing the V4 region of the *16S* rRNA gene for the fecal samples collected from mouse induction of kidney ischemia–reperfusion (I/R) [19]. Results showed that the bacterial community was dominated by increasing abundance of *Clostridium* and *Ruminococcus* and decreasing abundance of *Bifidobacterium* and *TM7* after I/R injury [36]. Another study demonstrated the hallmarks of I/R injury-induced dysbiosis mainly were increase of *Enterobacteriacea*, decrease of *Lactobacilli* and *Ruminococacceae*. Colonizing GF mice with post-AKI microbiota aggravated I/R injury severity with worsened inflammation in recipient mice compared to colonizing with microbiota from sham-operated mice [37]. Intestinal microbial dysbiosis is known as the consequence of AKI since several metabolites, including endotoxin and uremia toxins, are absorbed into the blood without clearing by the kidney [38].

Reportedly, serum lipopolysaccharide (LPS) level is a major indicator for verifying the occurrence of intestinal dysbiosis. Serum LPS circulates in the kidney, thus triggering inflammation and the oxidative stress pathway to promote kidney injury [39]. Low levels of endotoxemia and intestinal bacterial translocation were observed in mice with renal I/R injury [40]. Gut-derived uremic toxins (GDUT) consist of protein-bound toxins, p-cresyl sulfate (PCS), indoxyl sulfate (IS), and TMAO, with a detrimental effect on kidney function through the gut–kidney axis [41,42]. Furthermore, the serum level of GDUT and related metabolites affect kidney functions and are associated with an increased risk of kidney diseases [43]. With an increase in the abundance of bacteria that produce GDUT, a concurrent decrease was detected in the abundance of beneficial bacteria that produce SCFAs [44], which regulate the inflammation and energy metabolism in AKI. Moreover, many D-amino acids have been tested in the excrement of renal I/R mice, and only D-serine can be detected in the kidney [36]. These results indicated that the intestinal microbiota produces D-serine in response to AKI injury of renal I/R mice.

Previous studies have shown that intestinal dysfunction during kidney injury is due to the disruption of the intestinal barrier, which is partially mediated by the permeation of noxious molecules [40,45]. The key components that determine intestinal barrier function are the intestinal epithelial cells with tight intercellular junctions (TJs) [46]. Restoring epithelial TJ proteins *in vivo* reverses microbiota dysbiosis preventing toxin and pathogen translocation through intestinal barrier transported to the systemic circulation, which contributes to inflammation and progression of AKI [47,48]. In severe acute pancreatitis (SAP), tumor necrosis factor-alpha (TNF-α) elevates the intestinal permeability and promotes bacterial translocation from the epithelium, which further stimulates the excessive release of inflammatory cytokines and aggravates AKI [49]. During AKI sepsis, the disruption of the mucus membrane barrier may worsen systemic inflammation and potentiation of AKI [50]. Moreover, increased inflammatory cytokines disrupt the gut barrier function and increase the permeability by acting on the junctional complexes, such as apical TJs and junctional adhesion molecules (JAMs) [51] or *via* activation of myosin light chain kinase (MLCK) [52]. On the other hand, impaired clearance of water and metabolic products causes gut barrier injury and hyperpermeability [53].

Microbial stimuli influence the phenotype of renal lymphocytes and the expression of cytokines in kidneys and also modulate the outcome of AKI [20]. The equilibrium between Th1 and Th2 responses disrupts renal I/R injury, and GF mice exhibit abundant NKT cells and fewer Th2 cells with lower interleukin-4 (IL-4) levels than SPF mice [54]. Intestinal Th17 cells may migrate to the kidney through the S1P receptor pathway, thus promoting the development of autoimmune nephritis [55]. Also, Th17 cells play a major role in the chronic process of AKI induced by high salinity [56]. In renal I/R injury, AKI was associated with elevated Th17 and Th1 responses [57]. The depletion of microbiota significantly attenuated renal damage, maintained tubular integrity by reducing the maturation status of F4/80+ renal resident macrophages and bone marrow monocytes, as well as decreased the migratory capacity toward CX3CL1 and CCL2 ligands compared to the controls [58]. In addition, in kidney tissue after I/R, the activation of the costimulatory molecules CD80 and CD40 of bone marrow dendritic cells can be inhibited *via* products generated by the intestinal microbiota [59]. Previous studies demonstrated that GF mice are protected from intestinal IRI due to increased production of IL-10 [60]. The lack of intestinal microbiota is accompanied by IL-10-mediated inflammation, indicating the essential role of intestinal microbiota in facilitating acute renal inflammatory responses [61].

## Therapies targeting the intestinal microbiota in AKI

The composition, dynamics, and stability of gut microbiota are critical for diagnosing, treating, and preventing specific diseases. The gut microbiome transitioned from being a ‘missing’ organ to a potential target for therapeutic applications. Thus, targeting the intestinal microbiota might provide a novel therapeutic strategy in AKI.

### Probiotics and prebiotics

Probiotics are living microorganisms beneficial to the host health by improving the intestinal microbial balance, and the most studied probiotics are *Saccharomyces cerevisiae*, *Streptococci*, *Lactobacillus*, and *Bifidobacterium* species [62,63]. Prebiotics are usually defined as non-digestible carbohydrates, such as lactulose and fructooligosaccharides, that selectively stimulate the growth and activity of beneficial intestinal bacteria [64]. Hitherto, various probiotics and prebiotics have been widely popularized among the general public because of their robust therapeutic effects and fewer side effects [65]. These conventional treatments may be a valid alternative to drug-based therapy in the prevention or amelioration of intestinal microbiome-associated kidney diseases [66–68].

The present study suggested that preventive *Lactobacillus casei* treatment affects the inflammatory state by decreasing the expression of proinflammatory cytokines, TNF-α and IL-6, in an LPS-induced endotoxic AKI mouse model [69]. The administration of *Lactobacillus paracasei* significantly reduced the serum LPS levels in obese-insulin resistance-induced kidney injury model [70]. Typically, *Lactobacillus salivarius* BP121 suppresses the generation of uremic toxins and regulates AMPK- and TLR4-dependent TJ assembly, thus protecting against cisplatin-induced AKI by downregulating renal inflammatory mediators and decreasing the oxidative stress [71]. *Lactobacillus* mix addition also decreases the production of proinflammatory cytokines (TNF-α and IL-6) and prevents renal proximal tubular cell apoptosis in a cisplatin-induced renal injury in pig model [72]. Moreover, *Bifidobacteria* is another widely used probiotic along with *Lactobacilli* in kidney diseases [73,74]. *Bifidobacteria* pretreatment is a valid strategy to prevent intestinal barrier dysfunction and reduce bacterial translocation in mice following liver or intestinal I/R injury [75,76]. However, whether *Bifidobacteria* is beneficial to kidney I/R injury by improving microbiota dysbiosis is yet uncertain. A randomized controlled trial indicated that prebiotics is valuable in the modification of the stool microbiome with improved inflammatory indices during CKD.

Recently, the probiotic L. casei Zhang has been confirmed to slow the progression of acute and CKD through animal experiments and clinical trials [77]. The study showed that L. casei Zhang improved intestinal dysbacteriosis and elevated the useful metabolites (such as SCFAs and niacinamide) in mice AKI and CKD models. Also, oral L. casei Zhang postponed the decline in kidney function in patients with stage 3–5 CKD. Hitherto, only a few studies have discussed whether prebiotic supplementation is effective in AKI. Taken together, oral probiotics might alter the intestinal microbiome dysbiosis in AKI.

### SCFAs supplementation

SCFAs belong to fermentation metabolic end-products from complex polysaccharides produced by the intestinal microbiota and are comprised of butyrate, propionate, and acetate [78]. Moreover, the metabolic end-products are key candidate mediators for gut–kidney crosstalk. These metabolites also bind the G-protein membrane receptors (GPR) on intestinal epithelium or epigenetically modify histone deacetylase (HDAC) [79] and directly penetrate the epithelial membrane through transporter channels to affect kidney function [80]. The importance of SCFAs with respect to anti-inflammatory properties to protect against AKI has been utilized [81,82]. Treatment with SCFAs, especially acetate, inhibits inflammation and apoptosis processes and reduces NF-κB activation after kidney I/R injury [83]. The supplementation of dietary SCFAs in I/R AKI mouse model improves kidney dysfunction and protects from other complications [59]. Furthermore, SCFAs exert a strong influence and role *via* the immunomodulatory effects on the polarization of T-cell subsets through GPR expression [84]. In sepsis-induced AKI, SCFAs ameliorate immune function by inhibiting NADPH oxidase signaling in T cells [85]. SCFAs also upregulate serotonin (5-HT) [86], which modulates the immune homeostasis by either enhancing dendritic cell-mediated T-cell activation or affecting macrophage polarization and phagocytosis [87]. In addition, SCFAs are known as HDAC inhibitors [88] and can suppress high glucose-induced apoptosis of renal tubular epithelial cells by inhibiting HDAC2 due to its anti-oxidative property [89].

A recent study suggested that a high-fiber (HF) diet or SCFA supplementation prevents the development of AKI and subsequent CKD [90]. In experimental murine folic-acid nephropathy (FAN) mice models, HF improves the kidney function, such as less tubular injury on day 2 and less interstitial fibrosis and chronic inflammation on day 28 compared to normal chow-fed mice. Moreover, the study also found that the HF diet reduces AKI-induced dysbiosis, fosters the expansion of SCFAs-producing bacteria, and increases SCFA concentrations in fecal and serum. The main mechanisms are associated with HDAC inhibition and activation of GPR by SCFAs. However, studies have failed to report the application of SCFAs in clinical AKI patients. Overall, *in vitro* and *in vivo*, exogenous SCFA supplements may be a new therapeutic tool for preventing the progression of renal inflammation and the potential direction for translational clinical studies.

### Immunomodulator

With respect to the immune dysfunction related to the gut–kidney axis, dampening the immune response by targeting microbiota-derived mediators is an accepted strategy for limiting kidney damage. The animal models of kidney I/R injury showed that depletion of intestinal microbiota protected against AKI *via* reduced Th17, Th1 response, and expansion of Tregs and M2-polarized macrophages [57]. Th17 cell expansion and maintenance depend on the proinflammatory cytokine IL-23, which can be blocked with monoclonal antibodies against IL-23 [91] and IL-23/IL-12 [92]. In addition, IL-17A, secreted by Th17 cells, might be the most promising target and can be disrupted with monoclonal anti-IL-17A antibodies, such as secukinumab and ixekizumab [93]. Alternatively, monoclonal antibodies, such as brodalumab, can also inhibit IL-17 receptor signaling [94].

### Selective decontamination of the digestive tract (SDD)

SDD is an infection intervention strategy by topical administration of antibiotics to the oropharynx and the gastrointestinal tract, which was proposed about 30 years ago [95]. A recent study demonstrated that oral vancomycin reduces the levels of uremic toxins produced in the intestinal microbiota of CKD patients [96]. After hemodialysis, each participant took a single 250 mg capsule of vancomycin for 4 weeks. These results showed that the pre-dialysis mean plasma concentrations of both IS and PCS were elevated. However, following the administration of vancomycin, the IS and PCS concentrations decreased on day 2 or 5 and returned to baseline by day 28. Moreover, the primary change in the gut microbiome was the persistent decrease in diversity, which needs to be elucidated further.

Oral vancomycin not only disrupts the translocation of uremic toxins to the kidney by interfering with intestinal mucosal permeability but also decreases the amount of intestinal Th17 cells, which in turn alleviates renal inflammatory damage [55,97]. Several studies have shown that the addition of SDD reduces the infection rate and mortality in the intensive care unit (ICU), wherein AKI was a serious complication [98]. Thus, this is a promising method to break down the intestinal microbiota dysbiosis, followed by inhibition of inflammation and rebuilding the intestinal barrier [59,99]. However, recent studies have revealed that antibiotic-induced microbiome depletion (AIMD) disrupts host glucose homeostasis with reduced renal glucose and pyruvate levels, causing severe tubular injury after I/R [100]. Therefore, the rational application of antibiotics for the maintenance of intestinal flora homeostasis is challenging for AKI treatment.

### Other therapeutic approaches

The oral administration of spherical carbon adsorbent reduces the absorption of harmful metabolites through gastrointestinal sequestration. The oral administration of activated charcoal AST-120 upregulates the expression of the colonic epithelial TJ proteins, such as ZO-1, occludin, and claudin-1, which was associated with the intestinal epithelial barrier [101]. AST-120 reduces the level of IS-derived from tryptophan metabolism in plasma and uric levels and ameliorates CKD-induced endotoxemia and systemic inflammation [42,102]. Furthermore, CharXgen is a new activated and safe charcoal that lowers the level of protein-bound uremic toxins [103]. Thus, the application of carbon adsorbent might slow the chronic process of AKI.

Another study demonstrated the renoprotective effects of gut-derived D-serine in mediating AKI [36]. In a mouse kidney I/R model, AKI-induced gut dysbiosis contributed to the altered metabolism of D-amino acids, which decreased the activity of D-amino acid oxidase and increased the activity of serine racemase. In this study, oral D-serine administration repaired the kidney injury in this AKI mouse model. It also suppressed hypoxia-induced tubular damage during the early phase of injury and promoted post-hypoxic tubular cell proliferation, thereby deeming it as a potential novel therapeutic target for AKI.

In addition to the above drug treatment, traditional Chinese medicine (TCM) is gaining increasing attention to maintain intestinal microbiota balance [104]. Oral administration of high-dose of emodin (TCM extract) in gentamicin sulfate-induced AKI rats restores the disrupted microbiome compared to the control group [105]. Similarly, oral emodin protects the intestinal epithelial barrier and reduces uremic toxin accumulation in CKD patients [106]. Based on these results, TCM application in AKI might be one of the key therapeutic methods in the future.

### Future directions and perspectives

Fecal microbiota transplantation (FMT) has been explored as an effective and safe intervention for intestinal microbiota reprogramming [107]. It has also been shown to be a promising treatment in other diseases, including recurrent *Clostridium difficile* infection [108], neurological disorders [109], ulcerative colitis [110], and metabolic syndrome [111]. Interestingly, a recent study demonstrated that FMT alleviates tubulointerstitial inflammation in diabetic nephropathy rats by decreasing serum IL-6 levels. Furthermore, the desquamation and necrosis of tubular epithelial cells in the FMT group were significantly attenuated compared to the diabetic rats [112]. In addition, FMT has been confirmed to modulate renal phenotype and alleviate inflammation, including reduction of albuminuria immediately and a decreased expression of KC chemokine in the humanized mouse model of IgA nephropathy [113]. Another study indicated that FMT was efficacious in treating IgAN in two patients with refractory IgAN [114]. Strikingly, FMT treatment improved the gut microbiota disturbance in the adenine-induced CKD mice model, further limiting the accumulation of uremic toxins issued from the intestinal cresol pathway [115].

Intriguingly, the main pathological feature of AKI is characterized by acute tubular epithelial cells injury. Maladaptive or incomplete repair of renal tubules increases the profibrotic signaling leading to the transition of AKI to subsequent CKD [116]. Thus, the early use of FMT prevents renal function deterioration. However, the benefits and risks of FMT need to be assessed in clinical practice. A study reported that a patient died from infection because of drug-resistant *Escherichia coli* bacteria in donor stool samples [117]. The published literature on FMT in kidney diseases is limited; thus, future studies should explore this possibility in AKI [34]. Table 1 summarizes the recent advances in novel methods on intestinal microbiota for kidney diseases.

**Table 1.** Therapies targeting the intestinal microbiota in kidney diseases.

Methods	Detailed strain	Effects	Applications
Probiotics	*Lactobacillus, Bifidobacteria*	Anti-inflammatory, decrease oxidative stress and apoptosis, and limit absorption across intestine	LPS-AKI, CIS-AKI, CKD
Prebiotics	Lactulose	Stimulate the growth and activity of beneficial intestinal bacteria	CKD
SCFAs	Butyrate, propionate, and acetate	Modulate the immune homeostasis	IRI-AKI, Sepsis-AKI
Immune modulator	Monoclonal antibodies	Dampen the immune response	IRI-AKI
SDD	Vancomycin	Reduce uremic toxins, rebuild the intestinal barrier	AKI
Others	Charcoal adsorbent, D-serine, TCM	Protect the intestinal barrier, reduce uremic toxins	AKI, CKD
FMT	Intestinal microbiota reprogramming	Anti-inflammatory, repair tubular epithelial cells injury	CKD

## Conclusion

**Figure 1.**
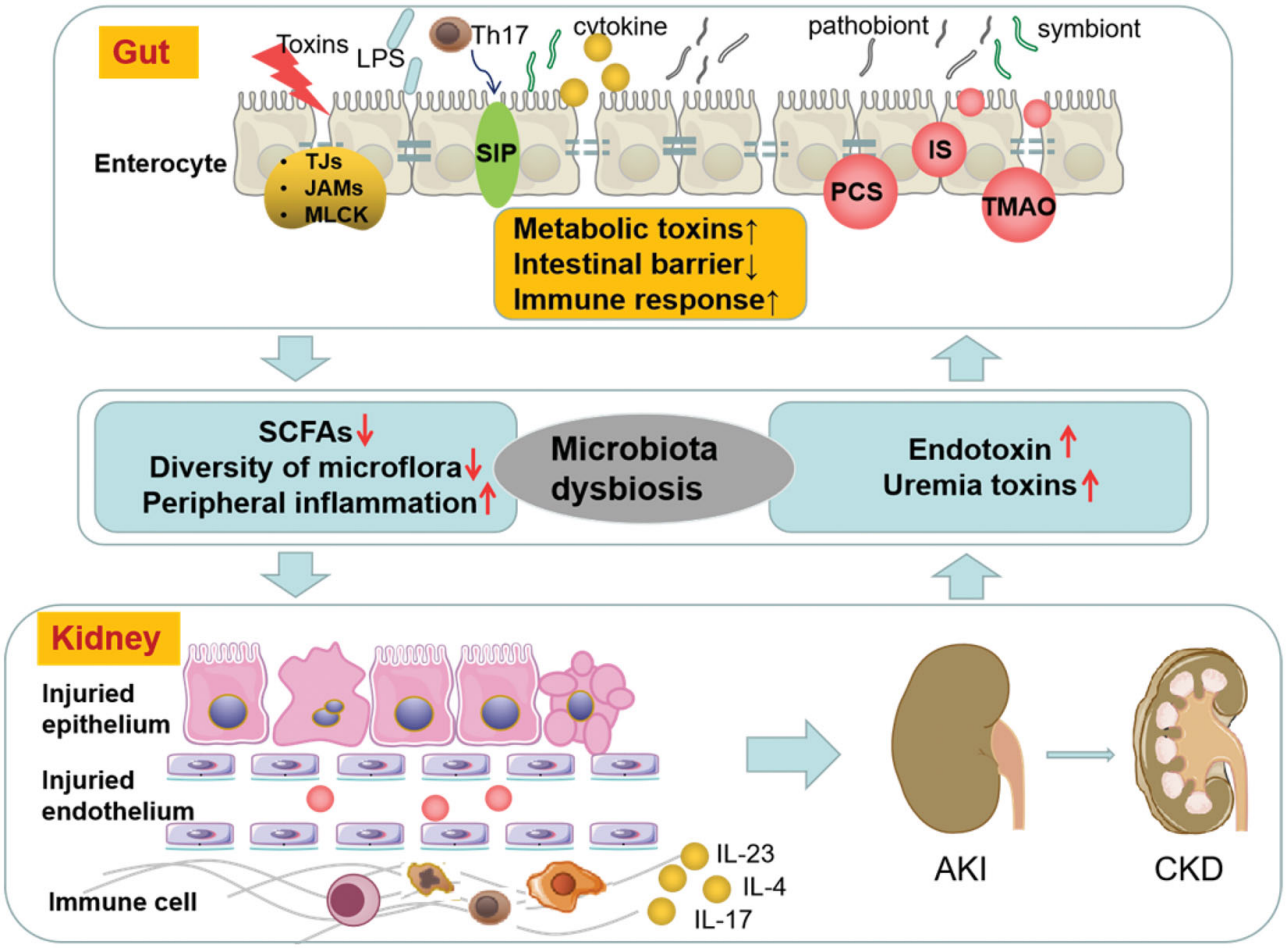
Vicious circle of AKI and intestinal microbiota dysbiosis. AKI profoundly alters enteric microbial compositional disruption, and the kidney can also be the direct target of intestinal microbiota dysbiosis by intestinal barrier disruption and excessive secretion of uremic toxins and triggers immune response. AKI profoundly alters enteric microbial compositional disruption, and the kidney is also the direct target of intestinal microbiota dysbiosis by intestinal barrier disruption and excessive secretion of uremic toxins and triggers immune response. (LPS: lipopolysaccharide; TJs: tight intercellular junctions; JAMs: junctional adhesion molecules; MLCK: myosin light chain kinase; SCFAs: short-chain fatty acids; PCS: p-cresyl sulfate; IS: indoxyl sulfate; TMAO: trimethylamine-N-oxide; AKI: acute kidney injury; CKD: chronic kidney disease).

The crosstalk of gut and kidney is a hot topic in human multiorgan diseases. A significant effort has been taken to understand the complex and abnormal correlation between AKI and intestinal microbiota dysbiosis to create a ‘vicious circle’ (Figure 1). AKI development alters enteric microbial compositional disruption, while the kidney can also be the direct target of intestinal microbiota dysbiosis by intestinal barrier disruption and excessive secretion of uremic toxins, which might trigger immune dysfunction that damages the renal tubular cells. Due to intestine microbiota dysbiosis’s critical role in AKI, novel preventive or therapeutic targets are under investigation. The present review summarized the treatment options on the regulation of intestinal microbiota in AKI, including the intake of probiotics and prebiotics, administration of beneficial intestinal metabolic products, such as SCFAs, the addition of the necessary modulator for microbiota immune, and selective decontamination of the digestive tract as oral antibiotics. In addition, activated charcoal, D-serine, and effective TCM are promising strategies to modulate intestinal microbiota in AKI. Despite the limited data available on FMT in kidney diseases, FMT might provide a reference for further research. To date, there is still a lack of clinical intervention trials investigating these novel AKI therapies. Therefore, further studies are urgently required to elucidate the underlying mechanisms and the clinical application of these treatments on intestine microbiota that would ameliorate AKI. Also, additional knowledge of intestinal microbiota-AKI is necessary as it may open new avenues for AKI therapy.
